# Supplementary material for: Polymer-free van der Waals assembly of 2D material heterostructures using muscovite crystals
Source: Nat Commun. 2026 May 4;17:6017. doi: 10.1038/s41467-026-72554-x (PMC13346611; doi:10.1038/s41467-026-72554-x)
Supplement: Supplementary file 1 — Supplementary Information [file 41467_2026_72554_MOESM1_ESM.pdf]

SUPPLEMENTARY INFORMATION: Polymer-free van der Waals assembly of 2D material heterostructures using muscovite crystals

Ian Babich,[1,2] Timofey M. Savilov,[1,2] Natalia A. Mamchik,[1,2] Kristina Vaklinova,[1] Nansi Zhou,[1,2] Denis S. Baranov,[1] Dmitrii A. Litvinov,[1,2] Virgil Gavriliuc,[1] Yue Yuan,[1] Amoz Chua,[2] Kenji Watanabe,[3] Takashi Taniguchi,[3] Mario Lanza,[1,2] Maciej Koperski,[1,2] Kostya S. Novoselov,[1,2] Alexey I. Berdyugin,[1,2,4,*] and Makars Šiškins[1,5,*]

[1] *Institute for Functional Intelligent Materials, National University of Singapore, 4 Science Drive 2, Singapore 117544, Singapore*
[2] *Department of Materials Science and Engineering, National University of Singapore, Singapore 117575, Singapore*
[3] *Advanced Materials Laboratory, National Institute for Materials Science, 1-1 Namiki, Tsukuba, 305-0044, Japan*
[4] *Department of Physics, Faculty of Science, National University of Singapore, Singapore 117551, Singapore*
[5] *School of Physics and Astronomy, University of Southampton, Highfield, Southampton SO17 1BJ, United Kingdom*

CONTENTS

Supplementary Note 1. X-ray photoelectron spectroscopy of bulk mica crystals 2

Supplementary Note 2. Atomic force microscopy of mica membranes used for transfer 2

Supplementary Note 3. X-ray photoelectron spectroscopy of heterostructures assembled using mica-assisted methods 3

Supplementary Note 4. Raman spectroscopy of heterostructures assembled using mica-assisted methods 6

Supplementary Note 5. Atomic force microscopy measurements of adhesion between mica and other 2D materials 7

Supplementary Note 6. Electron mobility comparison of mica-transferred graphene against state-of-the-art results from the literature 9

Supplementary Note 7. Additional characterization of 3R-MoS_2 sample. 9

Supplementary Note 8. Summary over fabrication conditions for the reported samples. 10

Supplementary References 10

* Corresponding authors:
alexey@nus.edu.sg, m.siskins@soton.ac.uk

Supplementary Note 1. X-RAY PHOTOELECTRON SPECTROSCOPY OF BULK MICA CRYSTALS

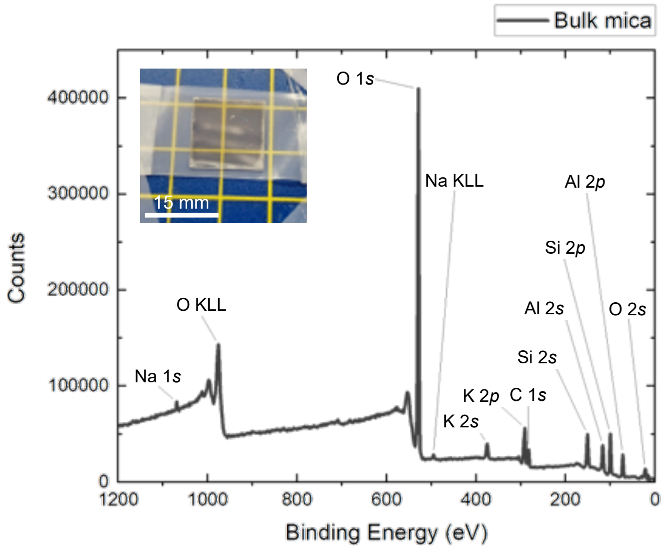

SUPPLEMENTARY Fig. 1. X-ray Photoelectron Spectroscopy (XPS) of bulk mica crystals. Peaks are labelled corresponding to their identified elements [1]. Inset: optical photo of the crystal.

Supplementary Note 2. ATOMIC FORCE MICROSCOPY OF MICA MEMBRANES USED FOR TRANSFER

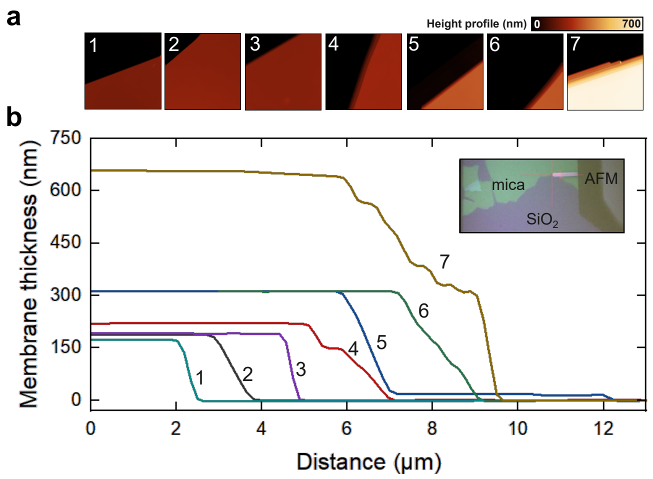

SUPPLEMENTARY Fig. 2. Atomic Force Microscopy (AFM) thickness measurements for some of the mica membranes and cantilevers discussed in the main text, purposefully placed on SiO_2/Si substrate. **a** AFM height profile scans of various mica membranes and cantilevers. **b** Measured membrane thickness of flakes from **a**. Inset: optical photo of the measurement.

Supplementary Note 3. X-RAY PHOTOELECTRON SPECTROSCOPY OF HETEROSTRUCTURES ASSEMBLED USING MICA-ASSISTED METHODS

To assess hypothetical contamination of resulting heterostructures assembled using mica-assisted transfer with mica leftover layers and particles, we performed local XPS measurements on multiple SLG/hBN devices. XPS spectra were collected with apertures between 25–50 μm, focusing the beam on the centre of the assembled heterostructures. Supplementary Fig. 3 and 4 present two representative measurements from independent hBN/SLG/hBN samples fabricated by mica-assisted stamping. The optical images (Fig. 3a and 4a) show the position of the XPS spot within the heterostructure. In both cases, no detectable signal from alkali elements characteristic of mica (Na, K) was observed (Fig 3b,c and 4b,c) [1]. Instead, clear B-N bonding signals corresponding to hBN were present (Fig 3d and 4d) [2], alongside C–C and C=C bonding features consistent with graphene (Fig 3e and 4e) [3]. Importantly, no Al 2p peak was detected (Fig 3f and 4f), confirming the absence of residual mica layers trapped in or on the stack [1].

As a control experiment, we performed identical measurements on an SLG/hBN heterostructure prepared using a mica cantilever method, where the mica layer was intentionally left underneath (Fig. 5a). In this case, The C–C and C=C features from graphene (Fig. 5d) were still present [3], while in addition to the K signal (Fig. 5b,c), a clear Al 2p signal from mica was also detected (Fig. 5e) [1]. These measurements demonstrate that mica-assisted assembly does not leave detectable mica residues in the heterostructure region in a result of transfer. In addition, the absence of Na, K, and Al peaks in assembled stacks confirms that the mica stamp can be used without introducing chemical contamination detectable by XPS.

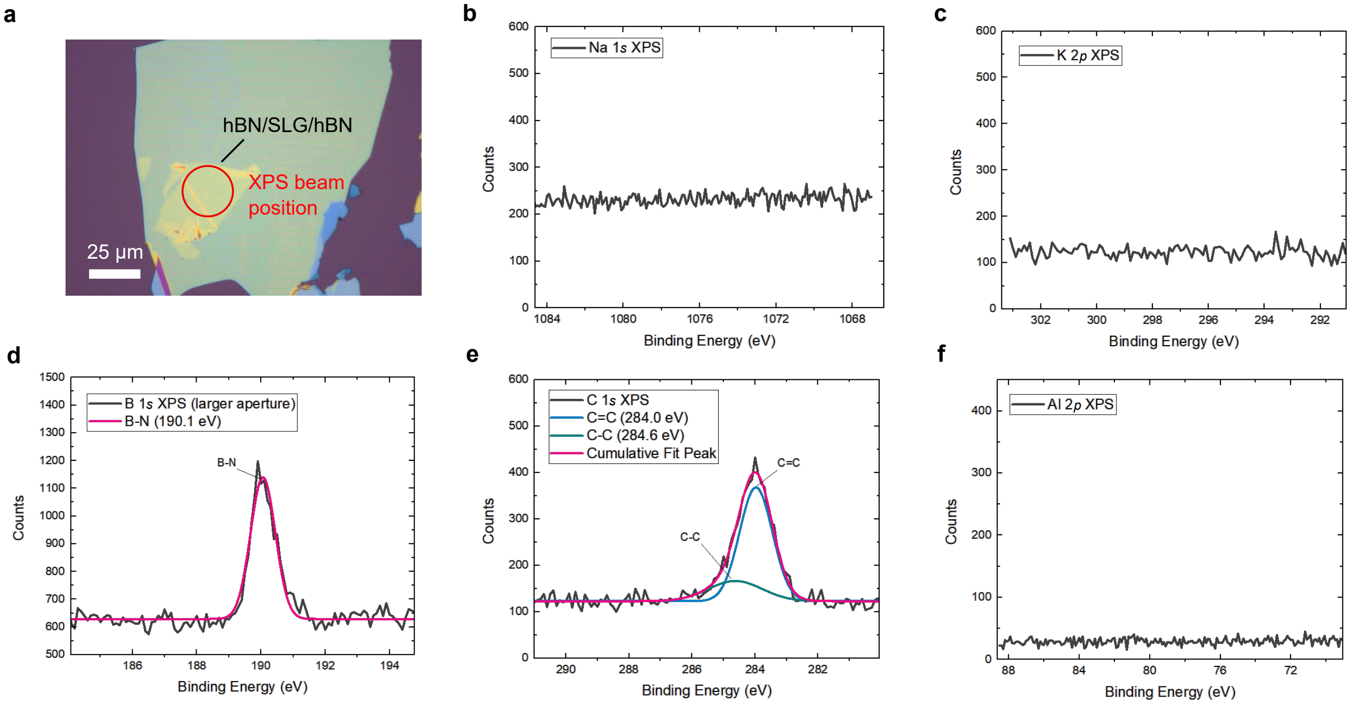

SUPPLEMENTARY Fig. 3. Local XPS experiment (sample 1, 25 μm aperture) on hBN/SLG/hBN heterostructure assembled using mica membrane-assisted method. **a** Optical image of the sample. **b** and **c** Absence of characteristic XPS peaks of typical ions of mica: Na (**b**) and K (**c**) [1]. **d** XPS peak corresponding to B-N bond of hBN in the heterostructure [2] (50 μm aperture). **e** XPS peak corresponding to C-C and C=C bonds of SLG in the heterostructure [3]. **f** Absence of characteristic XPS peak of Al in mica, indicating the absence of leftover mica layers in the heterostructure [1].

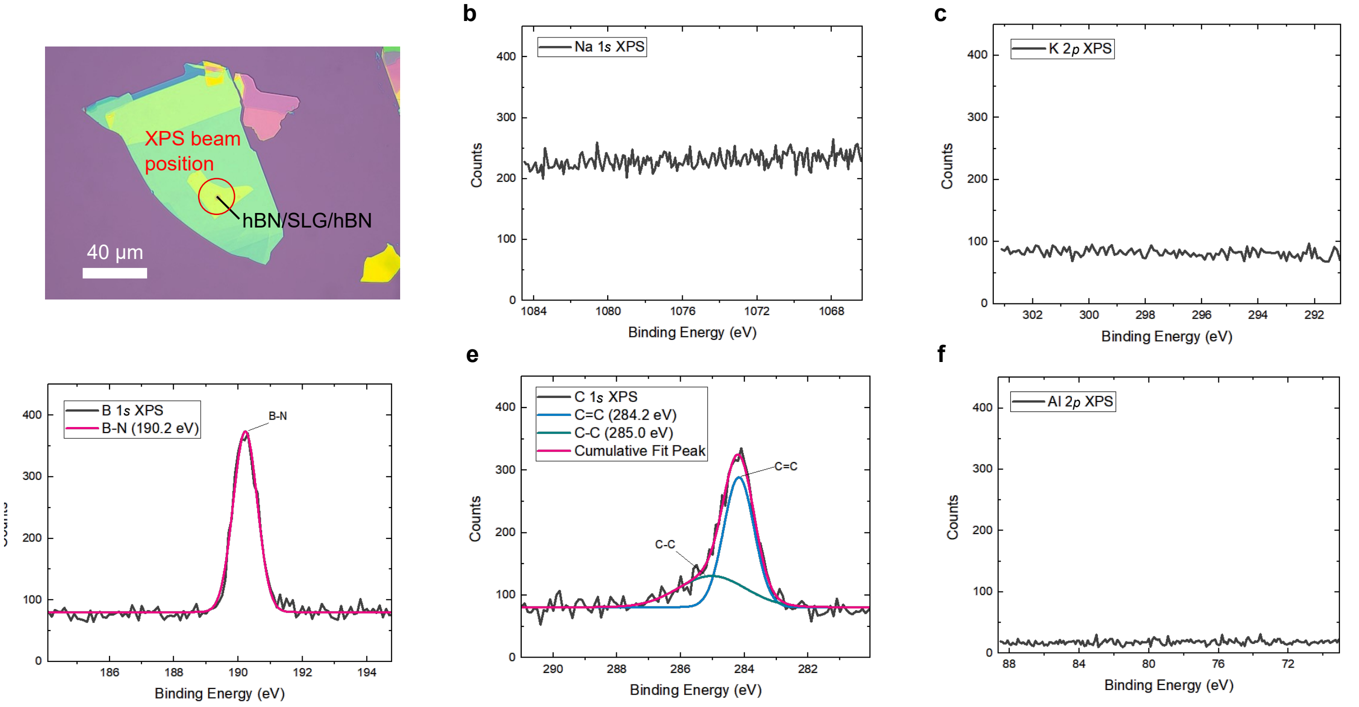

SUPPLEMENTARY Fig. 4. Local XPS experiment (sample 2, 25 μm aperture) on hBN/SLG/hBN heterostructure assembled using mica membrane-assisted method. **a** Optical image of the sample. **b** and **c** Absence of characteristic XPS peaks of typical ions of mica: Na (**b**) and K (**c**) [1]. **d** XPS peak corresponding to B-N bond of hBN in the heterostructure [2].**e** XPS peak corresponding to C-C and C=C bonds of SLG in the heterostructure [3]. **f** Absence of characteristic XPS peak of Al in mica, indicating the absence of leftover mica layers in the heterostructure [1].

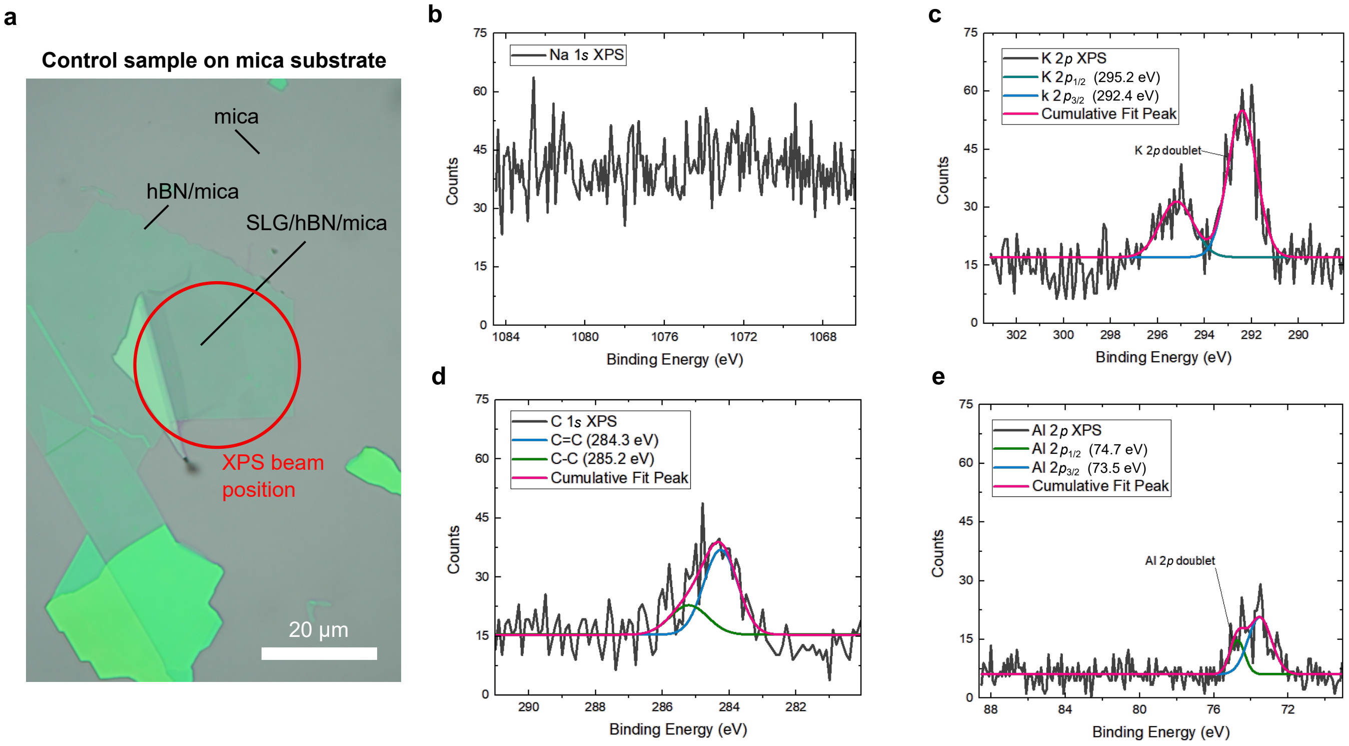

SUPPLEMENTARY Fig. 5. Local XPS experiment on the control sample, SLG/hBN heterostructure, assembled using mica-assisted method and released by flipping over and breaking off mica cantilever. **a** Optical image of the sample. **b** Absence of characteristic XPS peak of Na. **c** XPS duplet peak of K in mica [1]. **d** XPS peak corresponding to C-C and C=C bonds of SLG in the heterostructure [3]. **e** Characteristic XPS duplet peak of Al in mica, indicating the presence of mica layers in the heterostructure [1].

Supplementary Note 4. RAMAN SPECTROSCOPY OF HETEROSTRUCTURES ASSEMBLED USING MICA-ASSISTED METHODS

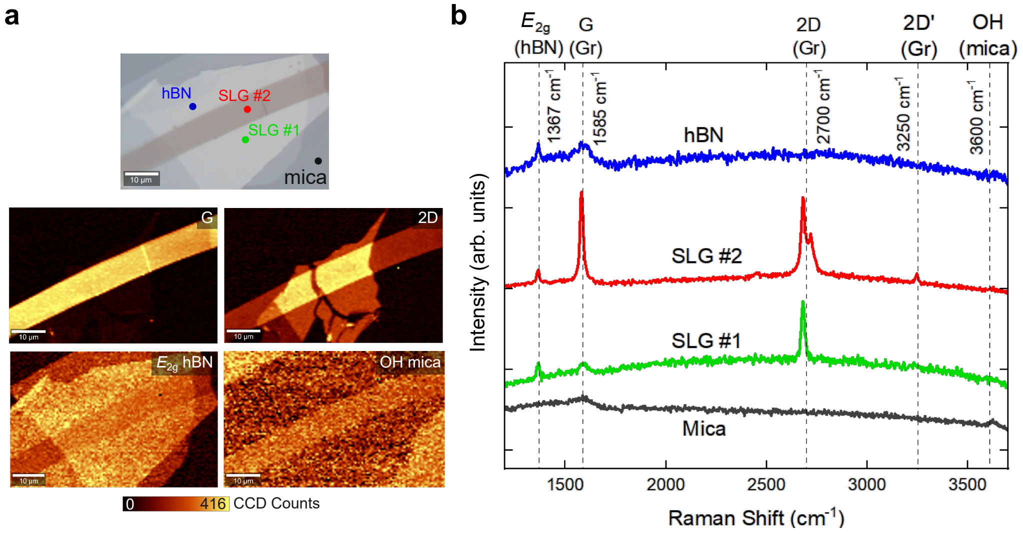

SUPPLEMENTARY Fig. 6. Raman spectroscopy characterisation of the SLG heterostructures from Fig. 2c, d of the main text. **a** Optical photo of the stack with corresponding Raman maps of graphitic peaks G and 2D, E_{2g} peak of hBN, and OH-group peak of mica. **a** Raman spectra of SLG and reference mica and hBN layers taken at points indicated by black, blue, green and red dots in **a**. Expected positions of characteristic peaks of mica, hBN and graphene are indicated with vertical dashed lines [4, 5].

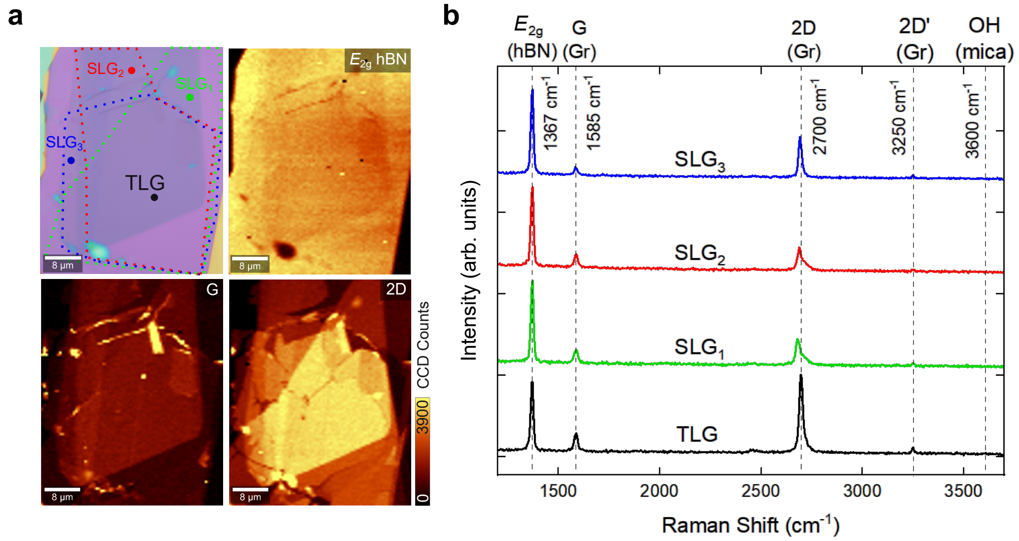

SUPPLEMENTARY Fig. 7. Raman spectroscopy characterisation of the TLG heterostructures from Fig. 2h, i of the main text. **a** Optical photo of the stack with corresponding Raman maps of E_{2g} peak of hBN, and graphitic peaks G and 2D. **a** Raman spectra of each SLG layer taken at points indicated by black, blue, green and red dots in **a**. Expected positions of characteristic peaks of mica, hBN and graphene are indicated with vertical dashed lines [4, 5]. OH-group peak of mica is completely absent.

Supplementary Note 5. ATOMIC FORCE MICROSCOPY MEASUREMENTS OF ADHESION BETWEEN MICA AND OTHER 2D MATERIALS

To quantitatively investigate how environmental factors influence the adhesion in mica-based transfer processes, we performed AFM force–deflection experiments, which provide a measure of the adhesion force between the cantilever and the substrate it adheres to [6]. These measurements quantify adhesion forces to representative materials (graphite, hBN, mica, and SiO_2) under varying humidity and temperature conditions. We first exfoliated mica cantilevers following the approach discussed in Fig. 1 of the main text. We coated mica cantilevers with Cr/Au (5/15 nm, single-sided) to improve the reflectance of their surface (see Supplementary Fig. 7a). The resulting coated mica cantilevers are mounted on a commercial Si AFM cantilever base by the adhesive tape. The structure is then mounted, tuned and calibrated similarly to commercial cantilevers on Bruker Dimension XR SPM (see Supplementary Fig. 7b). Using the thermal tune calibration, the resonance frequency of this mica cantilever is found to be 40.6 kHz with a Q-factor of around 58 (Supplementary Fig. 7c), which is comparable to soft commercial cantilevers. We calibrate the force channel using the standard procedure by measuring deflection sensitivity against the hard surface and fitting the resonance peak to the linear damped harmonic oscillator model.

A representative force–distance curve for mica–graphite contact is presented in Supplementary Fig. 7d, highlighting the characteristic pull-off feature upon retraction, the maximum value of which is related to adhesion force [6]. Systematic measurements across different substrates with equal peak contact force applied (Supplementary Fig. 7e) reveal that mica exhibits the strongest interaction with itself, followed by hBN, SiO_2, and graphite. As a control measurement, we performed two experiments for the mica-hBN pair at ambient and high humidity conditions, which we achieved by placing a wet cloth next to the sample together with a commercial household hygrometer. Supplementary Fig. 7e shows that mica–hBN adhesion is highly sensitive to ambient humidity: increasing relative humidity from 51% to $\sim$ 70% leads to a nearly order-of-magnitude increase in adhesion. This behaviour is consistent with enhanced interfacial water adsorption and capillary forces between surfaces, in line with previous observations for polymer-based transfer substrates [7, 8]. This crucial observation indicates the importance of control over humidity for the reproducibility of the fabrication recipes presented in the main text.

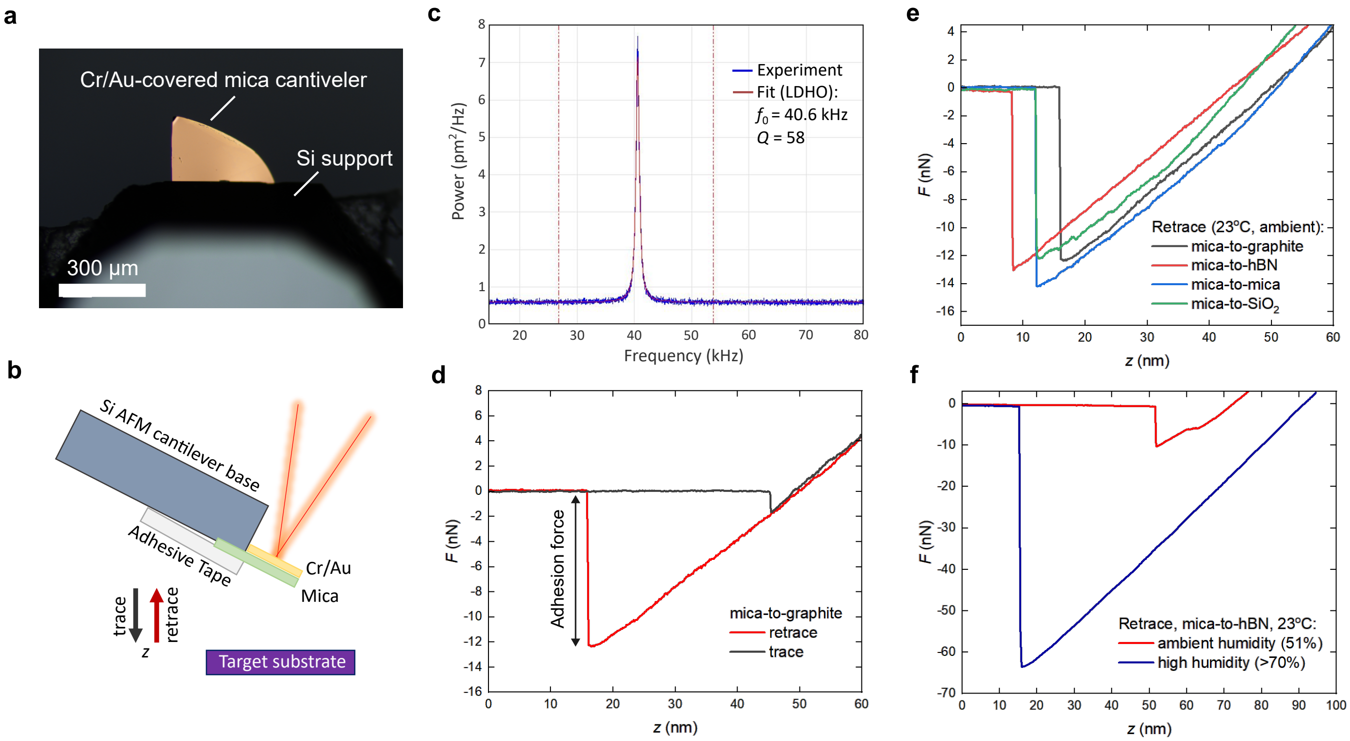

SUPPLEMENTARY Fig. 8. Adhesion force measurements with mica cantilevers. **a** Optical image of a Cr/Au-coated mica cantilever on a Si support. **b** Schematic of AFM force-deflection setup. **c** Resonance spectrum of a mica cantilever showing a fundamental mode at 40.6 kHz with $Q \approx 58$. **d** Representative force–distance curve for mica–graphite contact with adhesion force indicated [6]. **e** Adhesion forces between mica and graphite, hBN, mica, and SiO_2 at 23°C in ambient conditions. **f** Adhesion of mica-hBN at 23°C for two relative humidity levels, showing strong enhancement for high humidity.

Furthermore, we examined adhesion as a function of substrate temperature to quantify our empirically derived recipe from Fig. 1 of the main text, where an increase of temperature above $\sim 120°C$ improves the yield of heterostructure release from mica membranes and cantilevers onto bottom hBN flakes. Supplementary Fig. 9a shows force–distance curves for mica–hBN contacts acquired at increasing temperatures $30 - 110°C$. Adhesion decreases systematically with heating, approaching a plateau around $80 - 100°C$. This trend is summarised in Supplementary Fig. 9b, which plots adhesion forces for mica–graphite, mica–SiO_2, and mica–hBN as a function of temperature. All three systems show similar monotonic decreases with temperature, with a plateau at roughly $> 80 - 100°C$.

These results demonstrate that mica adhesion is both material-specific and strongly affected by environmental conditions. This highlights temperature as an important control parameter to tune adhesion between mica and 2D materials during heterostructure fabrication. It also shows that maintaining elevated substrate temperatures ($80 - 100°C$) is an effective strategy to stabilise adhesion forces outside inert atmospheres, where the precise control over ambient humidity is often not possible. We also note that these features are not limited to the mica method only, but may also be relevant to other van der Waals assembly methods where water adsorption contributes to variability of adhesion forces [7–10].

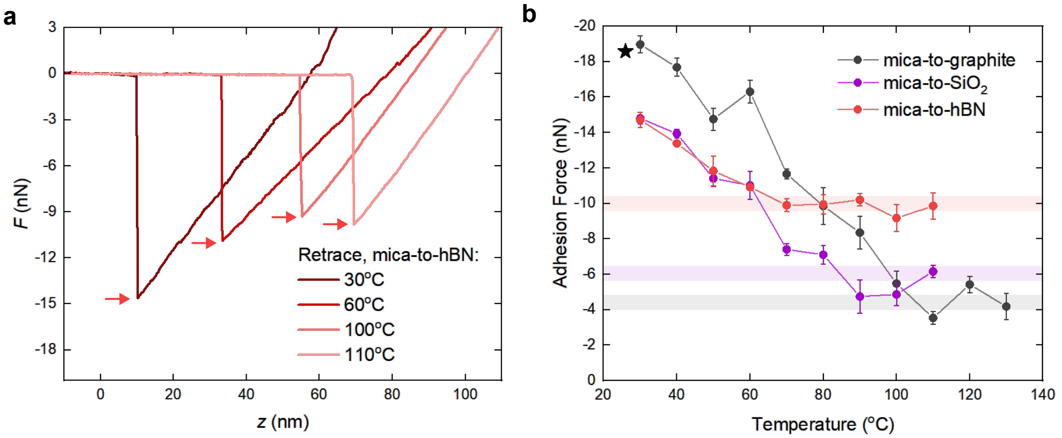

SUPPLEMENTARY Fig. 9. Temperature dependence of adhesion forces. **a** Force–distance curves for mica–hBN at 30, 60, 100, and 110°C. Red arrows indicate the change in adhesion force. **b** Extracted adhesion forces as a function of temperature for mica–graphite, mica–SiO_2, and mica–hBN. Coloured horizontal lines are a guide to the eye, indicating the saturation around $80 - 100°C$. Error bars are defined by the standard error around the mean of five consecutive measurements.

Supplementary Note 6. ELECTRON MOBILITY COMPARISON OF MICA-TRANSFERRED GRAPHENE AGAINST STATE-OF-THE-ART RESULTS FROM THE LITERATURE

SUPPLEMENTARY TABLE 1. Comparison of electronic performance for graphene Hall bar devices, fabricated using different state-of-the-art methods.

Fabrication method and sample	Device width (μm)	Mobility $\times 10^6$ ($cm^2V^{-1}s^{-1}$)	Mean free path (μm)	Onset for LL (mT)
Mica, SLG Sample 1	14	3-4	14	-
Mica, TLG Sample 2	4	1.5-2	4	5-6
Mica, SLG Sample 3	3.5	0.3	3.5	-
SiNx, SLG Sample 1 [9]	5	0.75	5	-
SiNx, SLG Sample 2 [9]	12.5	1.2	12.5	-
SiNx, SLG Sample 3 [9]	33.6	3.5	-	-
SiNx, SLG Sample 4 [9]	23	2.0	20	-
PC, BLG [11]	5	0.5	5	5-7
PC, SLG [12]	17	1	17	-
PC, SLG [13]	15	1	15	-
PC, SLG Sample 1 [14]	24	1.8	21	-
PC, SLG Sample 2 [14]	18	1.3	18	-
PC, SLG Sample 3 [14]	2.5	0.39	4	-
PC, SLG [15]	12	1	12	-

Supplementary Note 7. ADDITIONAL CHARACTERIZATION OF 3R-MOS$_2$ SAMPLE.

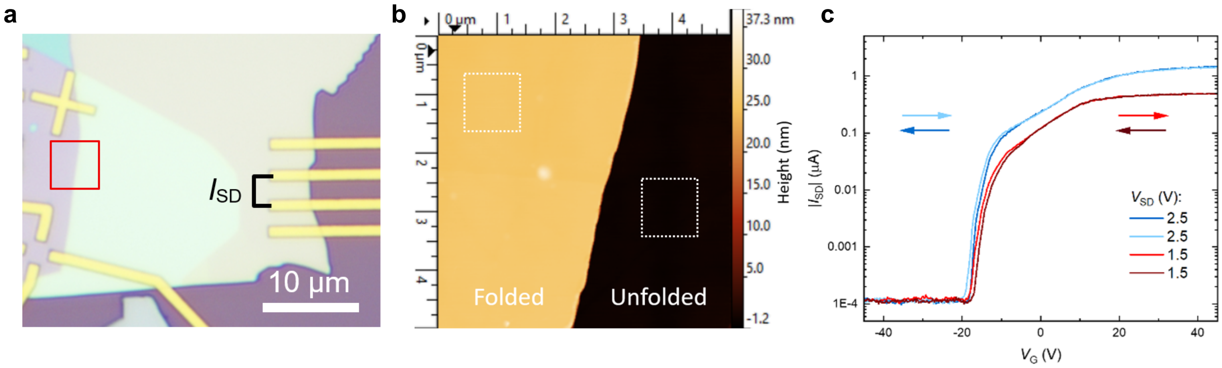

SUPPLEMENTARY Fig. 10. Additional characterisation of 3R-MoS$_2$. **a** Optical image of the sample with Ti/Au electrodes deposited. **b** AFM height map of the region indicated by the red box in **a**. Two dotted white line boxes indicate the area where surface roughness R_{pp} = 244 pm (folded) and R_{pp} = 182 pm (unfolded) was measured. **c** The drain current measured over the terminals indicated in **a** and Si as a gate electrode.

Supplementary Note 8. SUMMARY OVER FABRICATION CONDITIONS FOR THE REPORTED SAMPLES.

SUPPLEMENTARY TABLE 2. Fabrication conditions of reported samples.

Sample	Figures of the main text	Fabrication environment
Enc. SLG Sample 1	Fig. 2a-b	Ambient air
Enc. SLG Sample 2	Fig. 2c-d, 4g-i	Ambient air
Enc. SLG Sample 3	Fig. 4a-c	Glovebox (Ar, O_2/H_2O<0.1 ppm)
Enc. TLG Sample 1	Fig. 2g-i, 4d-f	Ambient air
hBN/hBN Sample 1	Fig. 3a-d	Ambient air
SLG/hBN Sample 1	Fig. 3h-l	Ambient air
SLG/hBN Sample 2	Fig. 5a-c	Glovebox (Ar, O_2/H_2O<0.1 ppm)
SLG/hBN Sample 3	Fig. 5d	Ambient air
SLG/hBN Sample 4	Fig. 5g	Ambient air
TBG Sample 1	Fig. 3m	Ambient air
TBG Sample 2	Fig. 3n-o	Ambient air
TBG Sample 3	Fig. 3p-q	Ambient air
3R-MoS_2 Sample 1	Fig. 5e	Ambient air
MoS_2 Sample 2	Fig. 5f	Glovebox (Ar, O_2/H_2O<0.1 ppm)
Enc. $CrBr_3$ Sample 1	Fig. 5h	Glovebox (Ar, O_2/H_2O<0.1 ppm)
$FePS_3$/hBN Sample 1	Fig. 5i	Glovebox (Ar, O_2/H_2O<0.1 ppm)

[1] Bhattacharyya, K. G. XPS study of mica surfaces. *J. Electron Spectrosc. Relat. Phenom.* **63**, 289–306 (1993).

[2] Feigelson, B. N. *et al.* Growth and spectroscopic characterization of monolayer and few-layer hexagonal boron nitride on metal substrates. *Nanoscale* **7**, 3694–3702 (2015).

[3] Nanri, Y. *et al.* Preparation of grafted few-layer graphene from K-THF-graphite intercalation compounds by the addition of aldehyde. *FlatChem* **24**, 100206 (2020).

[4] Neumann, C. *et al.* Raman spectroscopy as probe of nanometre-scale strain variations in graphene. *Nat. Commun.* **6**, 8429 (2015).

[5] Ben Aziza, Z., Zhang, Q. & Baillargeat, D. Graphene/mica based ammonia gas sensors. *Appl. Phys. Lett.* **105**, 254102 (2014).

[6] Rokni, H. & Lu, W. Direct measurements of interfacial adhesion in 2D materials and van der Waals heterostructures in ambient air. *Nat. Commun.* **11**, 5607 (2020).

[7] Ma, X. *et al.* Capillary-force-assisted clean-stamp transfer of two-dimensional materials. *Nano Lett.* **17**, 6961–6967 (2017).

[8] Cai, J., Chen, H., Ke, Y. & Deng, S. A capillary-force-assisted transfer for monolayer transition-metal-dichalcogenide crystals with high utilization. *ACS Nano* **16**, 15016–15025 (2022).

[9] Wang, W. *et al.* Clean assembly of van der Waals heterostructures using silicon nitride membranes. *Nat. Electron.* **6**, 981–990 (2023).

[10] Zomer, P. J., Guimarães, M. H. D., Brant, J. C., Tombros, N. & van Wees, B. J. Fast pick up technique for high quality heterostructures of bilayer graphene and hexagonal boron nitride. *Appl. Phys. Lett.* **105**, 013101 (2014).

[11] Babich, I. *et al.* Milli-Tesla quantization enabled by tuneable Coulomb screening in large-angle twisted graphene. *Nat. Commun.* **16**, 7389 (2025).

[12] Barrier, J. *et al.* Long-range ballistic transport of Brown-Zak fermions in graphene superlattices. *Nat. Commun.* **11**, 5756 (2020).

[13] Wang, L. *et al.* One-dimensional electrical contact to a two-dimensional material. *Science* **342**, 614–617 (2013).

[14] Purdie, D. G. *et al.* Cleaning interfaces in layered materials heterostructures. *Nat. Commun.* **9**, 5387 (2018).

[15] Huang, Z. *et al.* Mechanisms of interface cleaning in heterostructures made from polymer-contaminated graphene. *Small* **18**, 2201248 (2022).
