# Supplementary material for: Polymer-free van der Waals assembly of 2D material heterostructures using muscovite crystals
Source: Nat Commun. 2026 May 4;17:6017. doi: 10.1038/s41467-026-72554-x (PMC13346611; doi:10.1038/s41467-026-72554-x)
Supplement: Supplementary file 2 — Description of Additional Supplementary Files [file 41467_2026_72554_MOESM2_ESM.pdf]

Description of Additional Supplementary Files

File Name: Supplementary Video 1

Description: Optical microscope video recordings demonstrating the mica-assisted pick up, stacking, mechanical cleaning and release procedures. Process temperature and video playback speed are indicated for each process.
